# Role of diffusion-weighted MRI in differentiating benign from malignant bone tumors

**DOI:** 10.1259/bjro.20180048

**Published:** 2019-05-13

**Authors:** Anuradha Rao, Chandni Sharma, Raghuram Parampalli

**Affiliations:** Department of Radiology, Kidwai Memorial Institute of Oncology, Bangalore, Karnataka, India

## Abstract

**Objective:**

To evaluate the role of diffusion-weighted MRI in differentiating benign from malignant primary bone tumors. To know the sensitivity and specificity of diffusion weighted MRI and calculating apparent diffusion coefficient (ADC) cutoff in differentiating benign from malignant primary bone tumors.

**Methods and materials:**

This is a prospective observational study of 50 patients, who were clinically or radiologically suspected with primary bone tumor and referred to the Department of Radiodiagnosis, for radiography or for MRI. These patients underwent routine MRI sequences including diffusion-weighted MRI with *b*﻿-values of 0, 500 and 1000, followed by pathological examination supplemented by immunohistochemistry (wherever necessary). Hematological malignancies, recently biopsied cases and recurrent cases were excluded from the study.

**Results:**

Out of 50 patients with suspected bone tumors, 15 were benign (and tumor like lesions) and 35 were malignant primary bone tumors. The most common age group involved for both benign and malignant primary bone tumors was 11–20 years (23 cases—46%). In our study, total number of affected males were 27 (54%) and total number of affected females were 23 (46%) with M:F ratio of 1.17:1. In this study 72% lesions had appendicular bone involvement and 28% had axial bone involvement. 94.3% of malignant lesions showed restriction on diffusion-weighted imaging (DWI) and in 80 % of benign lesions restriction was absent on DWI which was statistically significant. Mean ADC levels in malignant lesions was 1.092 ± 0.497 and in benign lesions was 1.62 ± 0.596 which was statistically significant. Chondrosarcoma had highest ADC and Ewing’s sarcoma had lowest ADC values in malignant lesions. Chondroblastoma had highest ADC and Osteomyelitis had lowest ADC values in benign lesions. ADC value of 1.31 had highest sensitivity and specificity to differentiate between benign and malignant lesions.

**Conclusion:**

DWI is helpful in differentiating malignant from benign bone tumors and tumor like lesions with diffusion restriction favoring malignancy. Inspite of some overlap, ADC values of benign and malignant bone tumors are different and measurement of ADC values improves the accuracy of the diagnosis of bone tumors and tumor like lesions. Calculation of ADC may also be used as baseline reference to assess response to treatment in future or for follow up.

**Advances in knowledge:**

DWI imaging (and ADC values) has been extensively used in neuroimaging. Extension of this application to musculoskeletal–oncologic imaging is not so well studied. Apart from differentiating benign from malignant lesions which is the main focus of this study, assessment of response to treatment by ADC values may be possible in near future.

## Introduction

Evaluation of bone tumors involves a multimodality approach ranging from radiographs to cross-sectional imaging. The wide spectrum of the tumors of the bone, their diverse origin from multiple cell types along with tendency of these tumors to produce overlapping anatomic pattern sometimes make the final diagnosis of an osseous neoplasm, a challenge for the radiologist. Diffusion-weighted imaging (DWI) has advantages of short scanning time and does not need intravenous contrast administration. Few studies using DWI have shown promising results in musculoskeletal tumors. This study aims to evaluate the role of diffusion weighted MRI in differentiating benign from malignant primary bone tumors and proposing an ADC cutoff value in differentiating benign from malignant primary bone tumors.

## methods and materials

### Patients and design

About 50 patients who were clinically or radiologically suspected with primary bone malignancies and referred to the Department of Radiodiagnosis, Kidwai memorial institute of Oncology, Bangalore, India, were evaluated with diffusion-weighted MRI; followed by pathological examination supplemented by immunohistochemistry (wherever necessary) at a tertiary care oncology centre. The study period was from September 2016 to October 2017. Informed consent was obtained from all the patients enrolled in the study. Clinical history and examination findings were taken into consideration when needed. Patients having cardiac pacemakers, MRI incompatible prosthetic heart valves, cochlear implants or any metallic implants, claustrophobic patients, patients who could not lie down, dyspnoeic patients, patients with severe back ache, unco-operative patients, recently biopsied cases and recurrent cases were excluded from the study. Hematological malignancies involving the bone were excluded from the study. Initially radiographs were done for these patients with appropriate views according to the location of the lesion. If radiographs had been done recently before being referred to our hospital [Kidwai memorial institute of Oncology, Bangalore, India], those radiographs were obtained. CT scan was performed wherever necessary to correlate with the MRI findings.

### Image acquisition

Study was performed using 1.5 T Philips Achieva MRI machine. Appropriate body and extremity coils were used. The sequences used were axial *T*_1_, axial, coronal and sagittal *T*_2_ images, sagittal, coronal and axial proton density-weighted sequences images, post contrast fat sat *T*_1_W images in three planes, DWIs with *b*-values of 0, 500 and 1000. Patient's position (prone or supine) was determined by the area of abnormality. Body or surface coils were used according to the site of involvement. The smallest local coil that would adequately cover the anatomic area was used for imaging. The closest joint was included in the field of view in at least one plane to provide a landmark for surgical localization. The region of abnormality was positioned as close to the centre of the coil as possible. Prior to imaging the region of interest, a large field of view localizer using an increased diameter surface coil or body coil was used to accurately determine the proximal and distal extension of a large lesion. Slice thickness was 4–5 mm in most of the patients studied.

DWI was done for all patients before contrast administration, using multisection single shot spin echo-planar sequence (TR/TE/NEX: 2200/139 MS/1) with diffusion sensitivities of *b-*values = 0, 500 and 1000 s/mm^2^. Diffusion gradients applied sequentially in three orthogonal directions (X, Y and Z) with 5 mm slice thickness, interslice gap of 1 mm, field of view 240–400 mm and 128 × 256 matrix. Scanning time was about 120 s. The number of slices varied from one patient to another, and was chosen in a manner that covered the entire tumor with an extra slice in each direction. The trace images were obtained at different *b*-values: 0, 500 and 1000. Post-processing of DWI: four sets of DWIs for each section were obtained. ADC map corresponding to the average diffusion images where obtained. The circular or elliptical region of interest (ROI) was placed over the portions of the tumor which visually appeared to have the lowest ADC (assuming to correspond to the most cellular tissue), also attempting to include the largest area of tumor within the ROI (eliminating adjacent bone or soft tissues). The mean ADC values were obtained. In the tumors showing heterogeneous signal intensity, cystic areas within the tumor were avoided and at least three round ROIs (10–55 mm^2^) were placed on the ADC map corresponding to the areas of lowest ADC (on visual inspection). The position of the ROI was always checked with reference to conventional MRI.

### Image analysis

Two radiologists with 10 years and 20 years experience who were blinded with the clinical and other radiological information analyzed the images and calculated the ADC values.

On DWIs, the areas within the lesion which showed high signal (on high *b*-value images) with corresponding low signal on the ADC map were characterized as diffusion restricted areas. The imaging findings of MRI were correlated with pathological findings on histopathological examination (using appropriate staining methods) and immunohistochemistry. The final diagnosis was compared with first differential diagnosis on radiograph and MRI.

Patients included in our study were classified into two groups: benign and malignant.

### Statistical analysis

Statistical analysis was done by entering the data into Microsoft excel data sheet and was analyzed using SPSS 22 v. software (IBM SPSS Statistics, Somers, NY, USA). Categorical data was represented in the form of Frequencies and proportions. χ^2^ test or Fischer’s exact test (for 2 × 2 tables only) was used as test of significance for qualitative data. Graphical representation of data was done using MS Excel and MS word to obtain various types of graphs such as bar diagram and Pie diagram. *p*-value (Probability that the result is true) of <0.05 was considered as statistically significant after assuming all the rules of statistical tests. MS Excel**,** SPSS v. 22 (IBM SPSS Statistics, Somers, NY, USA) was used to analyze data.

## Results

50 patients with suspected bone tumor (15 benign, 35 malignant) were investigated with MRI including DWI, after taking their clinical history and relevant examination. The interpretations on MRI were correlated with the pathological findings [cytopathological, histopathological, Immunohistochemistry (wherever necessary)]

In current study, the most common age group involved for both benign and malignant primary bone tumors was 11–20 years (23 cases—46%), followed by 21–30 years (13 cases—26%). Minimal number of cases were found in age group of 0–10 years and >60 years (one case each—2%). Mean age of presentation was 24.8 years. In our study, total number of affected males were 27 (54%) and total number of affected females were 23 (46%) with M:F ratio of 1.17:1.

In the study majority of subjects were in the age group 11 to 20 years (46%), 26% were in the age group 21 to 30 years, 12% were in the age group 31 to 40 years, 8% in the age group 41 to 50 years, 4% in the age group 51 to 60 years and 2% in the age group 0 to 10 years and >60 years respectively.

In the study 72% lesions had appendicular bone involvement and 28% had axial bone involvement.

The commonest bones involved in the study were left and right femur (16% each respectively), followed by right humerus (12%), right and left tibia (8%) and others as shown in Table 1. On Radiographs majority (28%) were diagnosed to have Ewing’s sarcoma, 22% had Osteosarcoma, 14% had giant cell tumor (GCT).

**Table 1.** Bone involved in study subjects

Bone involved	Count	%
Right ala of sacrum	1	2.0%
Calcaneum	1	2.0%
Left femur	8	16.0%
Left fibula	3	6.0%
Left humerus	1	2.0%
Left inferior pubic ramus	1	2.0%
Left mandible ramus	1	2.0%
Left pubic bone	1	2.0%
Left radius	1	2.0%
Left scapula	1	2.0%
Left tibia	4	8.0%
Pubic bone	1	2.0%
Right femur	8	16.0%
Right hand	1	2.0%
Right humerus	6	12.0%
Right ilium	3	6.0%
Right ischium	1	2.0%
Right maxilla	1	2.0%
Right tibia	4	8.0%
Sacrum	2	4.0%

**Figure 1.**
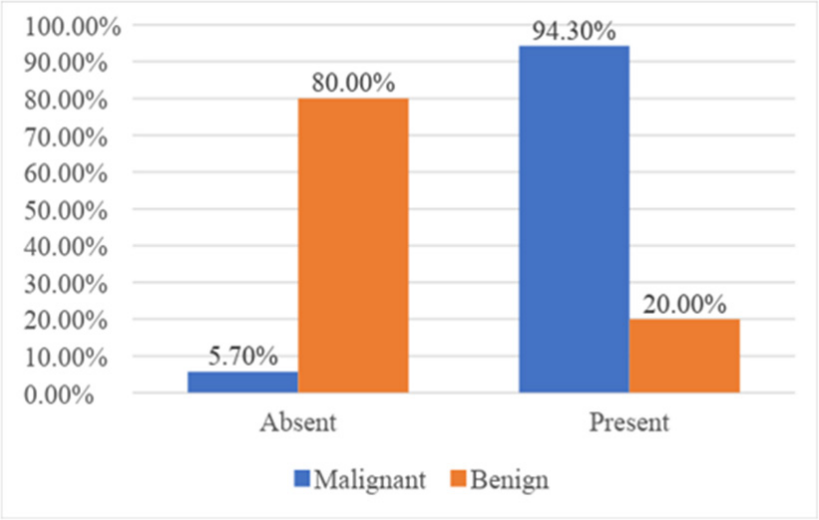
Bar diagram showing distribution based on morphology [diffusion restriction] of lesions on diffusion weighted image of MRI.

In 94.3% of malignant lesions diffusion restriction was present and in 80% of benign lesions had no diffusion restriction. This difference in DWI between malignant and benign lesions was statistically significant (Table 2, Figure 1).

**Table 2.** Distribution based on morphology of lesions on diffusion-weighted image of MRI

Diffusion restriction	HPE diagnosis					
	Malignant		Benign		Total	
	Count	%	Count	%	Count	%
Absent	2	5.7%	12	80.0%	14	28.0%
Present	33	94.3%	3	20.0%	36	72.0%

HPE, histopathological examination.

χ 2 = 1.020, df = 2, p =<0.001*

**Figure 2.**
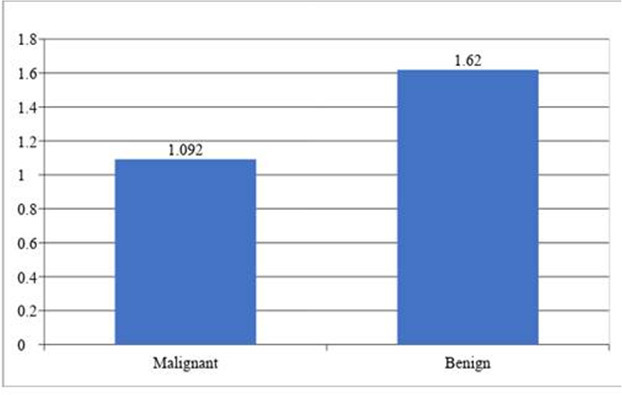
Bar diagram showing Mean ADC levels comparison between malignant and benign lesions. ADC, apparent diffusion coefficient.

The mean ADC in malignant lesions was 1.092 ± 0.497 and in benign lesions was 1.62 ± 0.596. This difference in mean ADC levels between malignant and benign lesions was statistically significant (Table 3, Figure 2).

**Table 3.** Mean ADC levels comparison between malignant and benign lesions

ADC	HPE diagnosis			
	Malignant		Benign	
	Mean	SD	Mean	SD
	1.092	0.497	1.62	0.596

ADC, apparent diffusion coefficient; HPE, histopathological examination.

χ 2 = 1.020, df = 2, *p* = 0.02*

Chondrosarcoma had highest ADC and Ewing’s sarcoma had lowest ADC values in malignant lesions. There was significant difference in mean ADC values with respect to different malignant lesions [Table 4].

**Table 4.** Mean ADC values with respect to different malignant lesions

Malignant lesions	HPE diagnosis		
	Malignant		
	ADC		
	No of subjects	Mean	SD
Ewing’s sarcoma	13	0.7	0.1
Osteosarcoma	11	1.1	0.4
GCT	3	1.1	0.1
Chondrosarcoma	5	2.1	0.1
Malignant fibrous histiocytosis	1	1.0	
Plasmacytoma	1	1.0	
Talengiectatic osteosarcoma	1	1.2	
*p*-value		<0.001*	

ADC, apparent diffusion coefficient; GCT, giant cell tumor; HPE, histopathological examination.

Chondroblastoma had highest ADC and Osteomyelitis had lowest ADC values in benign lesions. There was significant difference in mean ADC values with respect to different benign lesions [Table 5].

**Table 5.** Mean ADC values with respect to different benign lesions

Benign lesions	HPE diagnosis		
	Benign		
	ADC		
	No of subjects	Mean	SD
Enchondroma	3	2.1	0.1
Aneurysmal bone cyst	2	2	0.1
Chondroblastoma	2	2.2	0.6
GCT	2	0.9	0
Osteoid osteoma	2	1.5	0.1
Fibrous dysplasia	1	1.4	.
Non-ossifying fibroma	1	1	.
Osteochondroma	1	2.1	.
Osteomyelitis	1	0.5	.
*p-*value	0.008*		

ADC, apparent diffusion coefficient; GCT, giant cell tumor; HPE, histopathological examination.

ADC value of 1.31 had highest sensitivity and specificity to differentiate between benign and malignant lesions (Table 6).

**Table 6.** ROC curve showing ADC cut-off for benign and malignant lesions

Area under the curve				
Test result Variable(s): ADC				
Area	SE	*p-*value	Asymptotic 95% confidence interval	
			Lower bound	Upper bound
0.738	0.081	0.008*	0.579	0.897
ADC cut-off		Sensitivity		Specificity
0.53		1		0.057
0.851		0.933		0.371
1.31		0.733		0.771
2.39		0.067		1

ADC, apparent diffusion coefficient; ROC, receiver operating characteristic;SE, standarad error.

On MRI, majority, *i.e*. 26% were diagnosed to be Ewing sarcoma, 22% were Osteosarcoma, 12% were GCT.

On histopathological examination, majority, *i.e*. 26% were diagnosed to have Ewing sarcoma, 18% were Osteosarcoma, 10% were GCT and others as shown in Table 7.

**Table 7.** HPE diagnosis of bone lesions

**Diagnosis**		**Count**	**%**
Aneurysmal bone cyst		2	4.0%
Chondroblastic type of Osteosarcoma		1	2.0%
Chondroblastoma		2	4.0%
Chondrosarcoma		2	4.0%
Enchondroma		3	6.0%
Ewing sarcoma		13	26.0%
Fibrous dysplasia		1	2.0%
GCT		5	10.0%
Low grade chondrosarcoma		2	4.0%
Malignant fibrous histiocytosis		1	2.0%
Non-ossifying fibroma		1	2.0%
Osteochondroma		1	2.0%
Osteoid osteoma		2	4.0%
Osteomyelitis		1	2.0%
Osteosarcoma		9	18.0%
Osteosarcoma with secondary ABC changes		1	2.0%
Plasmacytoma		1	2.0%
Telangiectatic osteosarcoma		1	2.0%
**Type of lesion on HPE**	**Benign**	15	30.0%
	Malignant	35	70.0%

ADC, apparent diffusion coefficient; GCT, giant cell tumor; HPE, histopathological examination.

On histopathological examination, 30% of lesions were diagnosed as benign and 70% as malignant lesions.

## Discussion

Bone sarcomas account for 0.2% of all malignancies and the adjusted incidence rate for all bone and joint malignancies is 0.9 per 100,000 persons per year.^1^ The wide spectrum of the tumors of the bone, their diverse origin from multiple cell types along with tendency of these tumors to produce overlapping anatomic pattern make osseous neoplasm a highly challenging field from the radiologist’s point of view.

Evaluation of bone tumors involves a multimodality approach. Though the cross-sectional imaging has extraordinarily improved the ability to characterize tumors, the differential diagnosis of primary osseous neoplasm remains primarily based on their radiographic appearance. Radiographs provide information regarding lesion location, margin, matrix mineralization, cortical involvement and adjacent periosteal reaction.^2^ Conventional MRI is helpful in characterizing bone tumors, based on variations in the *T*_1_ and *T*_2_ relaxation properties of normal and pathologic tissue. However with overlapping signal characteristics of few of the benign/malignant neoplasms and non-neoplastic/reactive or inflammatory lesions, characterization of the pathology can be difficult even on conventional MR sequences. Also differentiating hyperintense tumor from reactive peritumoral edema can sometimes be challenging. Contrast MRI is helpful in defining the tumor margins and differentiates solid tumors from cysts, and identifying areas of necrosis. Contrast MRI may be of restricted use in patients with pregnancy, allergy to contrast material and in patients with renal failure due to the risk of nephrogenic systemic fibrosis.

DWI is a non-enhanced functional MRI technique that makes use of differences in the Brownian motion of water caused by variations in tissue microstructure. Quantitative measure of Brownian motion is indicated by the ADC. By using different *b* values trace ADC maps can be created on a pixel-by-pixel basis which gives quantitative analysis.^3^ Malignant lesions with highly cellular microenvironments limit diffusion and show low ADC values due to large number of cell membranes. Conversely high ADC values are observed in less cellular benign lesions due to free diffusion of water molecules. Thus DWI gives a quantitative functional assessment of cellularity at the molecular level. Short scanning time and the lack of need for intravenous contrast material are the other advantages which make it easy to be incorporated into a routine imaging protocol.^3,4^ However, DWI is most often referred for intracranial pathologies.^5^ Usage of DWI in the extracranial sites such as abdomen and pelvis is also encouraging and has become a routine sequence in oncologic settings.^6^ This feature can be made use of in differentiating benign and malignant lesions and assessing treatment.^4^

Wang et al in 2014 studied 198 lesions and inferred that the mean ADC value for benign bone tumors (1.17 ± 0.36×10^−3^ mm^2^/s) was significantly higher than that in malignant bone tumors (0.87 ± 0.20×10^−3^ mm^2^/s; *p* < 0.05) and with an ADC cut-off value ≥1.10 ×10^−3^ mm^2^/s the benign and malignant bone tumors could be differentiated with a sensitivity of 89.7%, a specificity of 84.5%, a positive predictive value of 82.6%, and a negative predictive value of 95.3%^7^

Kotb et al in 2014 studied 100 patients and concluded that malignant bone tumors have mean ADC values less than (1.31 × 10^−3^) mm^2^/s; while benign bone tumors have mean ADC values 1.43 × 10^−3^ mm^2^/s.^8^

Pekcevik et al in 2013 studied 26 patients and concluded that the mean ADC values from the area with lowest ADC values of benign and malignant bone tumors were 1.99 ± 0.57×10^−3^ and 1.02 ± 1.0×10^−3^ mm^2^/s, respectively and with cut-off value of 1.37 × 10^−3^ mm^2^/s, sensitivity was 77.8% and specificity was 82.4%, for distinguishing benign and malignant lesion.^9^ In their study an ADC value of 1.37 (×10^− 3^ mm^2^/s) was used for distinguishing benign from malignant lesions. Chondrosarcoma had highest ADC (2.99 × 10^− 3^ mm^2^/s) and Ewing’s sarcoma had lowest ADC (0.56 × 10^− 3^ mm^2^/s) values among malignant lesions.^9^

In the study conducted by Wang et al, the mean ADC value for benign tumors (1.17 ± 0.36 × 10^−3^ mm^2^/s) was significantly higher than that in malignant tumors (0.87 ± 0.20 × 10^−3^ mm^2^/s). An ADC cutoff value ≥1.10 × 10^−3^ mm^2^/s was able to differentiate between malignant and benign tumors.^7^

In the study conducted by Kotb et al, mean ADC levels in malignant lesions was 1.31 ± × 10^− 3^ mm^2^/s and in benign lesions was 1.43 × 10^− 3^ mm^2^/s.^8^

In the study by Eman et al, best cut-off criterion to differentiate benign and malignant tumors was ADC of ⩽0.67 with a sensitivity of 94%, specificity of 79% and accuracy of 87%.^10^

Shivani et al have concluded that benign lesions have higher minimum, and mean ADC values than malignancies [minimum (1.27 × 10^−3^ *vs* 0.68 × 10^−3^ mm^2^/s), mean (1.68 × 10^−3^ *vs* 1.13 × 10^−3^ mm^2^/s)] and concluded that Minimum ADC has the highest accuracy in discerning benign from malignant lesion.^11^

When compared with other studies, our study shows ADC cut-off values similar to the study done by Pekcevik et al (Table 8). In the study done by Pekcevik et al on 26 patients with similar MRI protocols as in our study for DWI imaging (with maximum *b* *﻿*-value of 1000); an ADC cut-off value of 1.37 (×10^− 3^ mm^2^/s) was suggested for distinguishing benign from malignant lesions. Chondrosarcoma had highest ADC (2.99 × 10^− 3^ mm^2^/s) and Ewing’s sarcoma had lowest ADC (0.56 × 10^− 3^ mm^2^/s) values among malignant lesions. Non-ossifying fibroma had lowest ADC 1.01 × 10^− 3^ mm^2^/s and bone cyst had highest ADC (2.72 × 10^− 3^ mm^2^/s) value, amongst the benign lesions in their study. More benign lesions were encountered than malignant lesions in their study. Our study was done on 50 patients with similar MRI protocols resulting in similar ADC cut-off value of 1.31 × 10^− 3^ mm^2^/s. In our study Chondrosarcoma had highest ADC (2.1 × 10^− 3^ mm^2^/s) and Ewing’s sarcoma had lowest ADC (0.7 × 10^− 3^ mm^2^/s) values among malignant lesions which is again is comparable to their study. Non-ossifying fibroma having least ADC value (1.0 × 10^− 3^ mm^2^/s) among benign lesions was again comparable to their study. We did not encounter a simple bone cyst in our sample. Chondroblastoma had highest ADC value (2.2 × 10^− 3^ mm^2^/s) in our study. The sensitivity and specificity of our study is almost comparable to their study. Our study had a better sample size of 50 (compared to 26 in their study) and more malignant lesions (35 malignant lesions and 15 benign lesions) than their study. Also our study considered only primary bone neoplasms. Metastasis was not included in our study. The study done by Pekcevik et al included both primary and metastatic neoplasms in the malignant group.

**Table 8.** Comparison of ADC values of benign and malignant lesions of present study with other studies

**Studies**	**Mean ADC for malignant lesions (×10^− 3^ mm^2^/s**)	**Mean ADC for benign lesions (×10^− 3^ mm^2^/s**)	**Cut-off ADC value (×10^− 3^ mm^2^/s**)	**Sensitivity % at cut-off ADC**	**Specificity % at cut-off ADC**
**Present study**	1.092 ± 0.497	1.62 ± 0.596	1.31	73.3	77.1
**Pekcevik et al ^11^**	1.02 ± 1.0	1.99 ± 0.57	1.37	77.8	82.4
**Wang et al ^8^**	0.87 ± 0.20	1.17 ± 0.36	1.10	89.7	84.5

ADC, apparent diffusion coefficient.

**Figure 3.**
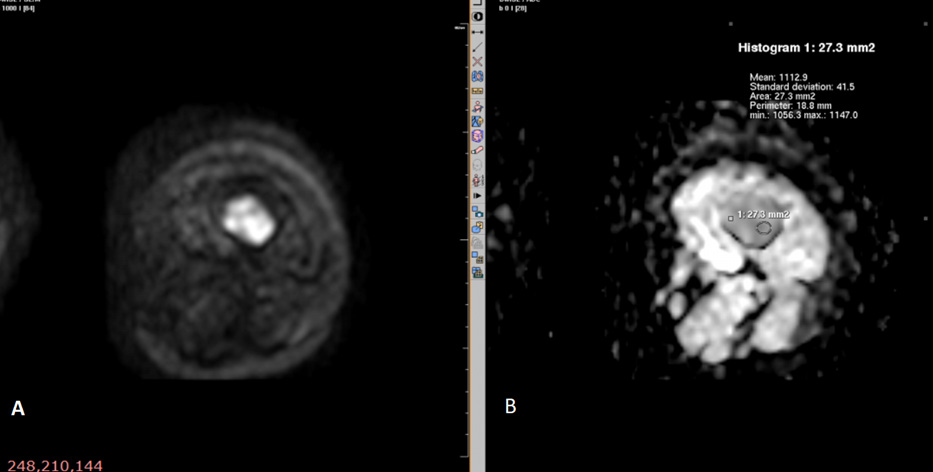
Diffusion-weighted MR sequence of osteosarcoma of left femur (axial section) showing diffusion restriction (A) and low ADC value of 1.1 (B).

**Figure 4.**
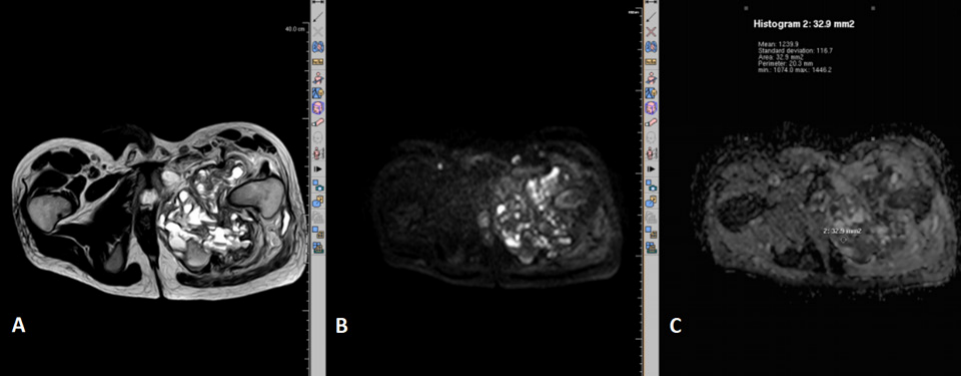
*T_2_* weighted (A) and diffusion-weighted MR sequence of pelvis (axial section) showing diffusion restriction (B) and low ADC value of 1.2 (C) in a large heterogenous lesion involving the left ischio-pubic bone. This was histopathologically proved as telangeictatic osteosarcoma. ADC, apparent diffusion coefficient.

**Figure 5.**
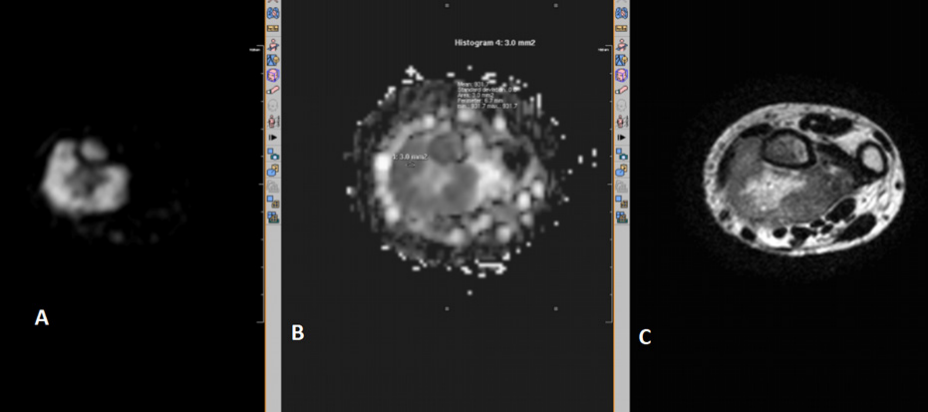
Diffusion-weighted MR image (A) and ADC map (B) of the left radius showing diffusion restriction with ADC value of 0.9. This was histopathologically proved to be giant cell tumor. ADC, apparent diffusion coefficient.

**Figure 6.**
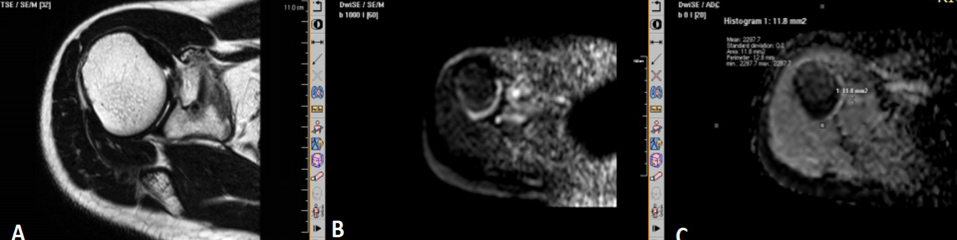
*T_2_* weighted MR image (A) showing a small hypointense focal lesion in the right scapula just adjacent to the glenoid. The lesion on diffusion-weighted image (B) and ADC map (C) shows an ADC value of 2.2. This patient had classical clinical symptoms of Osteoid osteoma, CT scan (not shown) showing typical nidus and was treated for the same. ADC, apparent diffusion coefficient.

In the present study, among malignant lesions, Diffusion restriction was present in 94.3% (Figures 3 and 4) and in 80% of benign lesions showed absence of diffusion restriction (Figures 5 and 6). Mean ADC levels in malignant lesions was 1.092 ± 0.497×10^− 3^ mm^2^/s and in benign lesions was 1.62 ± 0.596×10^− 3^ mm^2^/s.

**Figure 7.**
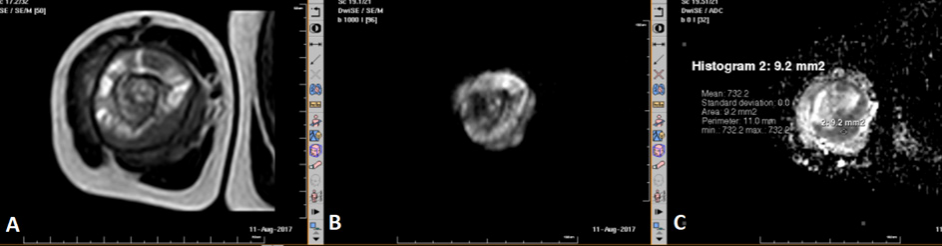
*T_2_* weighted (A) and diffusion-weighted MR sequence of right humerus (axial section) showing diffusion restriction (B)] and low ADC value of 0.7 (C). This was histopathologically proved as Ewing’s sarcoma. ADC, apparent diffusion coefficient.

Chondrosarcoma had highest ADC (2.1 × 10^− 3^ mm^2^/s) and Ewing’s sarcoma (Figure 7) had lowest ADC (0.7 × 10^− 3^ mm^2^/s) values among malignant lesions. Chondroblastoma had highest ADC (2.2 × 10^− 3^ mm^2^/s) among benign lesions. ADC cut off value of 1.31 × 10^− 3^ mm^2^/s had highest sensitivity and specificity to differentiate between benign and malignant lesions.

Nagata et al suggested that ADC values of myxomatous, cystic, and cartilaginous components were significantly higher than those of other tumors. In cartilaginous tumors, malignant tumor ADC values (2.33 ± 0.44) were higher than those of benign tumors (2.13 ± 0.13).^12^ The higher ADC values of chondrosarcomas, may be due to varying degrees of cellularity within a cartilaginous stroma which is likely to reflect relatively free extracellular water motion.^13^ Hence chondrosarcoma needs to be assessed separately and an ADC cutoff value with respect to chondrosarcoma mandates a detailed study in future with large number of chondrosarcomas.

The overlapping ADC values in benign and malignant lesions with respect to soft tissue tumors is explained by the fact that ADC values can be affected by cellularity and the extracellular matrix. The Myxoid matrix widely seen in the interstitial spaces in many soft tissue tumors can influence the ADC values resulting in significantly higher ADC values in myxoid tumors than non-myxoid tumors. In these tumors, it makes no difference if the tumor is benign or malignant.^14^ Our study had two chondrosarcomas with myxoid components.

Ewing’s sarcoma had lowest ADC values in our study (0.7 × 10^− 3^ mm^2^/s) which is in concurrence with study done by Pekcevik et al. Small round tumors are a group of undifferentiated aggressive embryonal tumors, and constitute a wide range of tumors including neuroblastoma, rhabdomyosarcoma, non-Hodgkin lymphoma, and the Ewing group of tumors.^9^ It is known that malignant lymphomas have characteristically low ADC values which have been explained by their high cellularity and nucleocytoplasmic ratio. Nagata et al have shown that soft tissue tumors belonging to this group have this property as well. According to them, these tumors tend to have lower ADC values and restricted diffusion than other malignant MSK tumors.^12^ Malignant non-small round cell tumors had significantly increased ADC when compared with small round cell tumors which was explained by the fact that small round cell tumors contain tissue with a relatively uniform population of small round cells, which have less extracellular space.^14^

Some of the malignant bone pathologies in this study exhibited low signal intensity on *T*_2_ weighted images and showed high signal intensity on DW images. These signal characteristics of these lesions are due to hypercellular solid components of the lesions which have increased nuclear/cytoplasmic ratios and also due to a reduction in the diffusion space of water protons in the extracellular and intracellular dimensions.

In addition, the low signal intensity on *T*_2 _weighted images and DW images of benign bone lesions, such as non-ossifying fibromas and osteofibrous dysplasia tumors, may have been due to the high density of fibers, low cellularity, and low water content in both the extracellular and intracellular spaces.

A high signal intensity on DW images of solid components with low ADC values can serve as a useful criterion for predicting malignancy in bone lesions, and that a low signal intensity on *T*_2_ weighted images and DW images of solid components with low ADC values may be an effective criterion for predicting the presence of benign disease.^7,15^

Out of the benign lesions, one case of osteomyelitis and two cases of giant cell tumors showed restriction on diffusion. Moderately vascularized network of round, oval, or spindle-shaped stromal cells and multinucleated giant cells are the histological features which probably decrease the extracellular space and result in decreased ADC values.^9,14^ One case of non-ossifying fibroma and fibrous dysplasia had relatively low ADC values of 1 and 1.4 respectively. These low ADC values could be due to the presence of abundant collagen-producing fibroblastic cells with dense network of collagen fibers within the extracellular matrix thus resulting in restricted the Brownian motion of water molecules. Wang et al have suggested that low signal intensity on *T*_2_ weighted images and low ADC values of benign bone lesions, such as non-ossifying fibromas and osteofibrous dysplasia tumors, may have been due to the high density of fibers, low cellularity, and low water content in both the extracellular and intracellular spaces and can be and useful criteria for predicting the benignity of the lesion.^7^

Differentiating solitary metastasis in a patient with known malignancy from benign tumors may be another application of DWI, which can be studied in future.

## Limitations

Being a tertiary care oncology setup, the number of malignant lesions encountered in the study were more than benign and inflammatory/infective lesions. Also since our institute is a government-funded tertiary care referral hospital and patients coming to our hospital are mainly from poor socioeconomic group, many of the patients have presented with advanced stage of the disease. The sample size of individual pathological category of tumors is low to extend the observed sensitivity, specificity, accuracy of MRI /DWI in determining pathological phenotype to a larger population. This study needs to be continued with larger sample size, with more representative samples in each subtype of tumors, also with probably further characterization into myxoid and non-myxoid varieties. The proposed value for the ADC threshold will need to be validated in a larger group of patients.

## Conclusion

DWI can be helpful in differentiating malignant lesions from benign lesions. Diffusion restriction on DWI favors malignancy. Even in patients with small lesions, DWI may be used along with routine sequences. Measurements of ADC values represent a method to differentiate tumors from tumor like lesions. DWI (and ADC values) may also be used in future as baseline study to assess response to treatment in future or for follow-up. Chondrosarcomas though being malignant show high ADC value, a fact which needs to be borne in mind while assessing the bone tumors.
